# Behavioral and neural bases of extinction learning in *Hermissenda*

**DOI:** 10.3389/fnbeh.2014.00277

**Published:** 2014-08-19

**Authors:** Joel S. Cavallo, Brittany N. Hamilton, Joseph Farley

**Affiliations:** ^1^Program in Neuroscience, Indiana UniversityBloomington, IN, USA; ^2^Department of Psychological and Brain Sciences, Indiana UniversityBloomington, IN, USA

**Keywords:** extinction, *Hermissenda crassicornis*, memory erasure, memory reversal, spontaneous recovery, reinstatement, invertebrate

## Abstract

Extinction of classical conditioning is thought to produce new learning that masks or interferes with the original memory. However, research in the nudibranch *Hermissenda crassicornis* (*H.c.*) has challenged this view, and instead suggested that extinction erased the original associative memory. We have re-examined extinction in *H.c.* to test whether extinguished associative memories can be detected on the behavioral and cellular levels, and to characterize the temporal variables involved. Associative conditioning using pairings of light (CS) and rotation (US) produced characteristic suppression of *H.c.* phototactic behavior. A single session of extinction training (repeated light-alone presentations) reversed suppressed behavior back to pre-training levels when administered 15 min after associative conditioning. This effect was abolished if extinction was delayed by 23 h, and yet was recovered using extended extinction training (three consecutive daily extinction sessions). Extinguished phototactic suppression did not spontaneously recover at any retention interval (RI) tested (2-, 24-, 48-, 72-h), or after additional US presentations (no observed reinstatement). Extinction training (single session, 15 min interval) also reversed the pairing-produced increases in light-evoked spike frequencies of Type B photoreceptors, an identified site of associative memory storage that is causally related to phototactic suppression. These results suggest that the behavioral effects of extinction training are not due to temporary suppression of associative memories, but instead represent a reversal of the underlying cellular changes necessary for the expression of learning. In the companion article, we further elucidate mechanisms responsible for extinction-produced reversal of memory-related neural plasticity in Type B photoreceptors.

## Introduction

Maladaptive learning is thought to be a major factor that underlies human anxiety disorders, such as phobias and post-traumatic stress disorder (PTSD; Garakani et al., 2006; Parsons and Ressler, 2013), as well as pathological conditions, including drug addiction (O’Brien et al., 1998; Di Chiara, 1999; Gass and Chandler, 2013) and eating disorders (Wardle, 1990; Jansen, 1998). Treatments for these disorders could therefore greatly benefit from an improved understanding of how unwanted associative memories are abolished. Extinction training has traditionally been used as one such method to reduce the magnitude of a conditioned response (CR). Extinction training involves the repeated presentation of a conditioned stimulus (CS) without the unconditioned stimulus (US), and results in the progressive reduction of the CR (Pavlov, 1927). Despite its name, extinction training rarely produces a permanent elimination of conditioning. Instead, conditioned associations may persist after extinction training, and the CR can return after the passage of time (spontaneous recovery; Pavlov, 1927; Myers and Davis, 2002), with a change in context (renewal; Bouton and Bolles, 1979), or following additional unreinforced presentations of the US (reinstatement; Rescorla and Heth, 1975). These phenomena have led to the hypothesis that extinction involves the formation of new learning that inhibits the original associative memory or competes with its expression (Rescorla and Cunningham, 1978; Robbins, 1990; Bouton, 1994; see Myers and Davis, 2002 for review). However, several lines of research (e.g., Delamater, 1996, 2004) suggest that this interpretation may be too limited. This contention is supported by recent evidence showing that modified extinction protocols can apparently permanently abolish associative learning. In these studies, extinction training delivered shortly after an isolated CS reactivates the original associative memory and is believed to initiate reconsolidation processes that update the original memory with new information (i.e., the CS does not signal the US; Monfils et al., 2009; Schiller et al., 2010). This technique appears to eliminate the behavioral changes produced by associative fear conditioning in rodents (Monfils et al., 2009; Rao-Ruiz et al., 2011; Flavell et al., 2011, but see Chan et al., 2010; Ishii et al., 2012) and humans (Schiller et al., 2010; Steinfurth et al., 2014, but see Golkar et al., 2012; Kindt and Soeter, 2013), and reverses some of the underlying cellular changes associated with fear conditioning (e.g., AMPA GluR1 receptor insertion) (Clem and Huganir, 2010). In other cases, unmodified fear extinction in rats produces similar behavioral and cellular reversal effects when administered within specific time intervals following learning acquisition (e.g., 1 h) (Mao et al., 2006; Myers et al., 2006, but see Maren and Chang, 2006; Woods and Bouton, 2008). Thus, under specific (not yet fully understood) circumstances, extinction can apparently involve the “destabilization” or “erasure” of associative memories on the behavioral and cellular levels.

The nudibranch mollusk *Hermissenda crassicornis* (*H.c.*) has proven to be an important model system for studying the cellular and molecular processes that underlie learning and memory because of its simple and tractable nervous system (~20,000 neurons), capacity for associative learning (Alkon, 1974; Farley, 1985; Britton and Farley, 1999; see Blackwell and Farley, 2009 for review), and the ability to localize sites of neural plasticity that are causally related to memory acquisition and storage (Farley et al., 1983). *H.c.* exhibits several cardinal features of vertebrate associative conditioning, including excitatory classical/Pavlovian conditioning (Farley and Alkon, 1980, 1982; Farley, 1987a), pairing- and stimulus-specificity (Farley and Alkon, 1982; Grover et al., 1987), contingency learning and partial-reinforcement during acquisition (Farley, 1987a,b), potentiation of (excitatory) conditioning by discrete stimulus compounds (Farley et al., 1997; Farley and Jin, 1997), superior learning for distributed vs. massed training trials (Farley and Alkon, 1987; Farley, 1987b; Rogers et al., 1994; Muzzio et al., 1999), sequential- and temporal-order sensitivity of CS-US pairings (Grover and Farley, 1987; Matzel et al., 1990), conditioned inhibitory (CI) learning (Britton and Farley, 1999; Walker et al., 2010), partially distinct mechanisms for short-, intermediate-, and long-term forms of memory (Crow et al., 1997; Epstein et al., 2003), and savings effects and latent memory following forgetting (Matzel et al., 1992). However, despite the extensive research conducted using *H.c.*, only one study has investigated the extinction of excitatory conditioning.

Richards et al. (1984) found that extinction training appeared to erase/destabilize associative memories in *H.c.* that are formed using repeated pairings of light (CS) and high-speed rotation (US) (see Farley, 1988b; Blackwell and Farley, 2009; Crow, 2004 for reviews). This associative conditioning procedure produces suppression of phototaxis (CR), a behavior that can be extinguished using repeated light-alone presentations (Richards et al., 1984). Although phototactic suppression was readily extinguished in *H.c.*, it did not show spontaneous recovery when measured 24 h after conditioning. The absence of spontaneous recovery was interpreted as possible evidence that extinction had erased the original associative memory (Richards et al., 1984). However, it remains unclear whether the associative memory would show spontaneous recovery at later intervals, or be observed using reinstatement procedures. Richards et al. (1984) also showed that extinction training reversed the pairing-produced increases in excitability (input resistance, peak and steady-state light response) of synaptically-isolated ocular Type B photoreceptors (Richards et al., 1984), which are a principal site of memory storage (Farley and Alkon, 1982; Richards and Farley, 1987) that are causally linked to conditioning-produced phototactic suppression (Farley et al., 1983). Richards et al. (1984) recorded extinction-produced changes in excitability using synaptically-isolated Type B cells in order to establish that B cells were an intrinsic site of plasticity for extinction training, rather than a passive synaptic consequence of changes occurring elsewhere. However, because synaptically-isolated (axotomized) Type B cells are unable to produce action potentials, it was not possible to determine whether pairing-produced increases in B cell spike frequencies (Farley and Alkon, 1982; Farley, 1987a) were also reversed by extinction training. This is a critical point, since it is alterations in light-evoked spike frequencies (rather than generator potentials *per se*) that are most directly related to downstream changes in the sensory-motor circuits that control phototaxis and other light-modulated behaviors (e.g., “clinging”) changed by associative learning (Farley and Alkon, 1982; Goh et al., 1985; Crow, 2004).

When the preceding behavioral and neural results from *H.c.* suggesting that extinction erased/destabilized memory were first reported, they were largely without precedent and stood in marked contrast to the prevailing wisdom that extinction produced new learning that masked and/or interfered/competed with expression of the original memory, without substantial alteration or eradication of the original memory. Additionally, the molecular understanding of memory formation and storage in *H.c.* was at a relatively early stage in 1984, and mechanisms that might contribute to extinction-produced erasure/destabilization of original memory were unknown. However, the recent resurgence of interest and research concerning the possibility that extinction may destabilize/eradicate/erase, or more generally allow for “updating” and editing of original memory (see Quirk et al., 2010; Auber et al., 2013; Flavell et al., 2013, for reviews), raises the possibility that extinction-produced erasure/destabilization may not be as exceptional for *H.c*. as originally thought. And, since recent research in *H.c.* points towards a suggestive commonality in the signaling pathways that underlie CI learning- and extinction-produced decreases in B cell excitability (Walker et al., 2010; Farley et al., submitted), we felt that a re-appraisal of the phenomenon of extinction in *H.c.* was in order.

Therefore, we have taken several approaches to further investigate whether associative memories in *H.c.* are detectable on the behavioral and cellular levels after extinction training. First, we extended the findings of Richards et al. (1984) and obtained multiple measures of phototactic behavior following extinction training at 2-, 24-, 48- and 72-h retention intervals (RIs) to look for evidence of spontaneous recovery. Second, we examined whether additional unsignaled rotation stimuli given after extinction training would reinstate extinguished phototactic suppression. Third, we studied the effects of varying interval length between learning acquisition and extinction training (15 min vs. 23 h) on the persistent reduction of the CR. Finally, we characterized the effects of extinction training on the light-evoked spike frequencies of Type B photoreceptors.

## Methods

### Animals

Adult *H.c.* were provided by Monterey Abalone Co. (Monterey, CA) and individually housed in perforated 50-ml plastic tubes in aquaria containing artificial seawater (ASW, Bio-sea Marine Mix, AquaCraft, Hayward, California, pH 7.8–8.2) at 15°C on a 6.5/17.5-h light/dark cycle with a radiant intensity of 4 μW/cm^2^, as previously described (Richards et al., 1984). Animals were fed with scallops (*Mytilus edulis*) twice weekly, but food was removed 48 h prior to behavioral testing.

### General behavioral training

**Figure 1:**
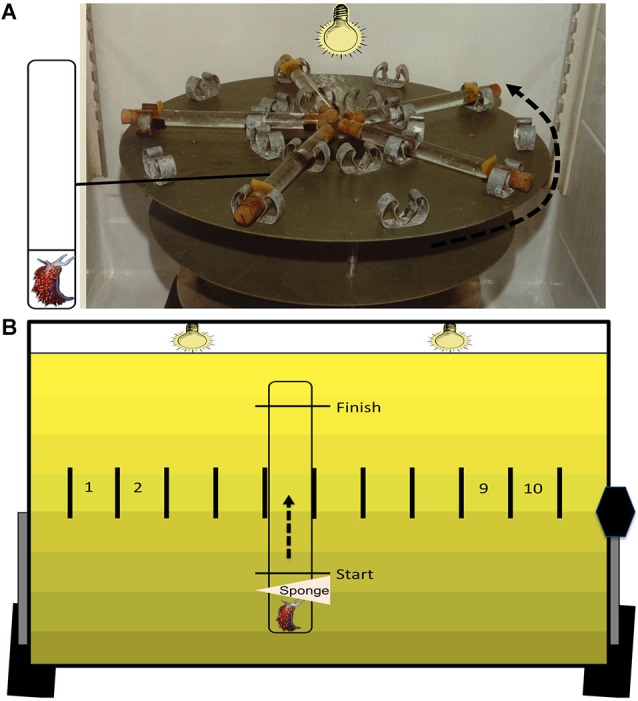
**Training and testing apparatus**. The training apparatus **(A)** is a high-speed (100 RPM) turntable. On the surface are 10 pairs of radially arranged clips. Each pair of clips secures one plastic tube that is filled with ASW and corked at both ends. The inset depicts each tube containing two compartments with flow-through holes and one animal that is confined to the small compartment. During associative conditioning, animals are given 50 presentations of simultaneous light (30 s) and rotation (30 s) with a 2 min variable inter-trial interval. The testing apparatus **(B)** is used to assess phototactic behavior, before and after training. Animals are placed in single-compartment plastic tubes that are filled with ASW. Animals are confined to one end using a sponge stuck into the tube through a small hole. Markings on each tube denote the start and finish line, and each tube is clipped into one of 10 individual testing lanes. During dark adaptation, the surface of the testing table is oriented horizontally. At the start of testing, the table is tilted upright (43° angle) and the two testing lights are turned on, which results in a gradient of illumination on the testing table (see Section Methods for further details). The sponges are removed, and the latencies to initiate locomotion (start latency) and to cross the distal finish line (finish latency) are recorded for each animal.

The methods and apparatus used for behavioral training have been described previously (Farley, 1987a) and methods for extinction training were designed after Richards et al. (1984). Animals were placed into clear plastic tubes filled with ASW and mounted on a turntable in a refrigerator (11°C) (Figure 1A). Vestibular stimulation consisted of high-speed rotation (100 RPM), while photoreceptors were stimulated using a 60 W incandescent light source (intensity of 56 μW/cm^2^) located above the turntable. The duration and timing of stimuli were controlled automatically using an IBM computer running DOS connected to a digital/analog controller made and programmed by engineers at Indiana University Department of Psychological and Brain Sciences. Animals were randomly assigned to one of six treatment conditions: *Untrained*, *Paired*, *Random*, *Immediate* *Extinction* (*Imm-Ext*), *Delayed Extinction* (*Del-Ext*), or *Delayed Extended Extinction* (*Del Exd-Ext*). The *Untrained* animals received no behavioral training and remained in the home aquarium during the scheduled training session. The *Paired* animals received two consecutive daily sessions of paired conditioning (50 trials each). One trial consisted of paired light (30 s) and rotation (30 s) presentations (simultaneous onsets and offsets), with an inter-trial interval (ITI) of 2 min (variable). *Random* animals received the same amount and type of training as paired conditioning (same stimulus duration and ITI), but had randomly presented light and rotation stimuli, thus serving as a control for non-associative phototactic suppressive effects of training stimuli. Animals in the *Paired + Extinction* group (referred to hereafter as just *Imm-Ext* or *Del-Ext* animals) received 2 days of paired conditioning followed by either immediate (15 min interval) or delayed (23 h interval) extinction training, which consisted of 25 light alone presentations (2 min inter-stimulus interval, ISI). *Del Exd-Ext* animals received three sessions of 50 light-alone presentations (24 h intervals) starting 23 h after acquisition training. After paired training, animals from the *Imm-Ext* group had the ASW in their tubes exchanged and were placed back into the turntable training apparatus. Animals were given 15 min of dark adaption followed by extinction training and then returned to their home aquarium. *Del-Ext* and *Del Exd-Ext* animals were returned to their home aquarium after paired conditioning and given extinction training the following day (23 h after the end of paired conditioning). All animals were trained in the middle of their light cycle, from ~10 am–2 pm and dark adapted for 15 min prior to any conditioning.

### Behavioral testing

Behavioral testing was conducted as described previously (Richards et al., 1984). Animals were placed in ASW-filled testing tubes and restricted to one end using a sponge during the dark adaptation period. The behavioral testing apparatus consisted of a flat surface with ten lanes, each with a secured testing tube (Figure 1B). Two light bulbs (25 W) were located 48.5 cm above the testing lanes and shielded by a metal reflector to emit a light gradient with low intensity (15 μW/cm^2^) at the bottom of the tubes (i.e., the start), and higher intensity (600 μW/cm^2^) at the top (i.e., the finish). The testing surface was horizontally positioned during dark adaptation and tilted upright at a 43° angle at the start of testing to maximize phototaxis due to negative geotaxis (see Farley and Alkon, 1982; Grover et al., 1987). After dark adaptation, the testing light was turned on, the table was tilted upright, and sponges were removed. *H.c.* phototaxis was assessed by recording the latencies to start and finish locomotion along the length of the tube (11.6 cm) at four RIs relative to the end of paired conditioning: 2-, 24-, 48-, and 72-h. These latency values were converted to a suppression ratio (SR) score using the equation, $\left(\frac{Before}{Before+After}\right)$ where the Before and After scores are the latency values (in seconds) before and after conditioning. An animal showing no change in phototaxis would have a SR of ~0.5, while animals with suppressed phototaxis would have a SR of <0.5. Cut-off scores of 25 min were assigned in tests in which animals failed to move past the start line (for start latency) or reach the finish (finish latency). See Farley and Alkon (1980, 1982) for further discussion of this metric for quantifying changes in *H.c.* phototactic behavior. Animals with pre-conditioning (baseline) finish latency scores greater than 15 min (~5% of animals) were excluded from further study.

### Nervous-system preparation

General methods for preparation and electrophysiological recordings from the *H.c.* nervous system have been described extensively in previous publications (e.g., Farley and Alkon, 1982; Farley, 1987a,b). The dissected *H.c.* circumesophageal nervous system was secured on a microscope slide within a 495 μL well of tank ASW (15°C). Each isolated nervous system was incubated in protease (1 mg/ml; Subtilisin A, Sigma P5380) for ~8–9 min at room temperature (~18–20°C) to facilitate cell impalement. After incubation, nervous systems were rinsed with ten volumes of cold (4°C) standard ASW composed of the following (in mM): 430 Na^+^, 10 K^+^, 10 Ca^2+^, 50 Mg^2+^, 10 Tris HCl/Tris Base, and 570 Cl−, pH = 7.6–7.8.

### Intracellular recording

Intracellular recordings from isolated nervous systems were obtained using previously described methods (Farley and Alkon, 1987; Farley, 1988a). Glass microelectrodes (A and M Systems, catalog No. 6020) filled with 1.5 M KCl (40–50 MΩ) were used to impale Type B photoreceptors. A silver/silver chloride wire connected the electrode solution to the head stage, and was used for the ground electrode. All recordings were made with an Axoclamp 900 A (Axon Instruments) amplifier and appropriate head stages. Signals were PCM-digitized using a Digidata 1440 recorder (Axon Instruments) and stored on a Windows computer using pCLAMP 10 software (Molecular Devices, Sunnyvale, CA). All recordings were obtained at room temperature (~18–20°C) in standard ASW, and were done without knowledge of the conditioning history of the preparation.

### Data analysis

The degree of phototaxis was assessed at each RI for both start and finish latencies using a SR score (see Behavioral Testing section above). Several measures of Type B cell excitability were obtained using pCLAMP 10 software, including: resting membrane potential (*V*_m_), input resistance (*R*_in_), and light-evoked spike frequency (determined by measuring the action potentials, in Hz, during the last 10 s of the 30 s LS). The input resistance was calculated using Ohms law. A brief (200 ms) current pulse of −0.25 nA was given and the voltage change recorded. Type B cells with *V*_m_ more positive than −39 mV and *R*_in_ less than 30 MΩ were considered damaged and were discarded.

### Statistical analysis

Differences in behavioral SRs were determined using repeated measures analysis of variance (ANOVA) and Bonferroni *post hoc* pairwise comparisons. Differences in Type B cell spike frequencies during LSs 1 and 2 were determined using a one-way ANOVA and Bonferroni *post hoc* tests. All statistics were performed using SPSS 20.0 software. In cases where a Mauchly’s test of sphericity revealed a violation of sphericity (*p* <0.05), a Greenhouse-Geisser correction was used. One-tailed significance tests were used and significant *p* values are <0.05.

## Results

### No evidence for spontaneous recovery after extinction training

**Figure 2:**
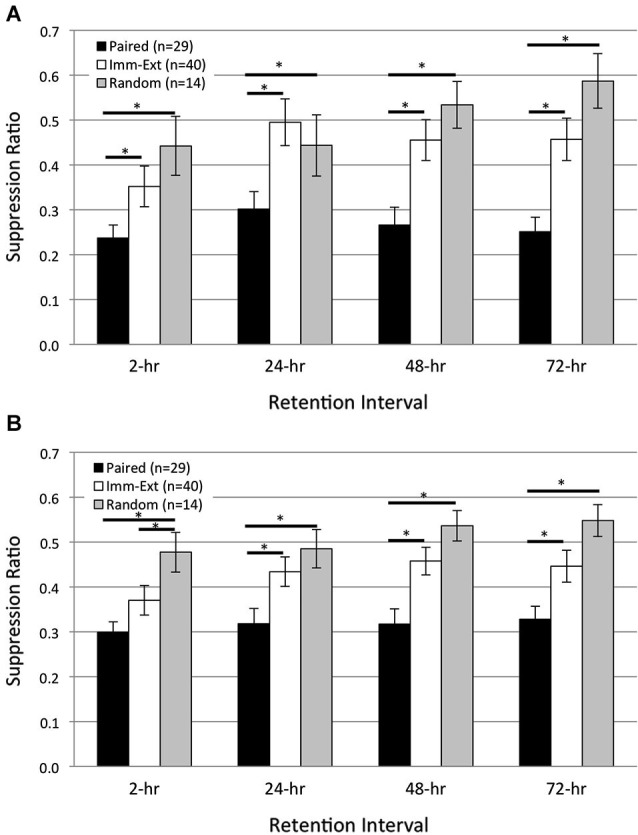
**Immediate extinction training attenuates pairing-produced phototactic suppression and returns phototactic behavior back to control (pre-pairing) levels, without evidence of spontaneous recovery**. Summary data for start **(A)** and finish **(B)** latency suppression ratio (SR) scores measured at four different retention intervals (RIs) post-acquisition. Start and finish latency values were converted to SR scores for each animal (see Section Methods). Animals given paired training showed significant phototactic suppression (i.e., smaller SR scores) compared to animals given random training (non-associative control group) at all four RIs for both start **(A)** and finish **(B)** latency behavior. Immediate extinction training (*Imm-Ext* group) given 15 min after paired conditioning greatly weakened the pairing-produced phototactic suppression and resulted in phototactic behavior that was similar to baseline control values. *Imm-Ext* animals showed significantly less phototactic suppression (greater SR scores) than Paired animals at the 2-h RI for start **(A)**, but not finish **(B)** latency behavior, and significantly less phototactic suppression at the 24-, 48-, and 72-h RI for both start **(A)** and finish **(B)** latency behavior. Spontaneous recovery of pairing-produced phototactic suppression was not observed at any RI following immediate extinction training. The post-extinction SR scores of *Imm-Ext* animals were significantly different from those of *Random animals* at only the 2-h RI and only for finish latency behavior. No other differences were found at any of the remaining RIs for both start **(A)** and finish **(B)** latency behavior. Error bars are ± S.E.M and significant *p* values (*p*’s < 0.05) are denoted by an asterisk.

The spontaneous recovery of a CR following extinction training is generally taken as evidence of an enduring associative memory (see Myers and Davis, 2002 for review). Contrapositively, if no spontaneous recovery is observed following extinction (assuming sensitive testing methods have been used) then an enduring associative memory is not present, or is at least undetectable. To establish whether spontaneous recovery of pairing-produced phototactic suppression occurred after extinction, phototactic behavior (start and finish latencies) was measured before associative conditioning and at four RIs, 2-, 24-, 48-, and 72-h post acquisition training. Separate groups of animals were given pairings of light and rotation (*Paired* group) alone, or pairings followed shortly (15 min) by extinction training (*Imm-Ext* group). A third non-associative control group received light and rotation presentations in a random fashion (*Random* group). The results indicated that pairings produced phototactic suppression that was reversed by extinction training back to control levels. Phototactic suppression did not spontaneously recover at any RI (Figure 2), which suggested that the memory for associative conditioning had been severely weakened, or possibly erased, by extinction training.

After training, *Paired* (*n* = 29) animals moved more slowly than *Random* (*n* = 14) animals and showed greater phototactic suppression (i.e., greater start and finish latencies and smaller suppression ratio, SR, scores). These findings are consistent with prior *H.c.* research, and indicate that phototactic suppression is pairing-specific (Farley and Alkon, 1980, 1982; Richards et al., 1984). Across all RIs, *Paired* SR scores were significantly smaller compared to the *Random* group for both start (Figure 2A; *F*_(1,21)_ = 9.68, *p* = 0.003) and finish (Figure 2B; *F*_(1,21)_ = 14.30, *p* = 0.001) latency behavior. Extinction training attenuated the phototactic suppression produced by paired training and analysis over all RIs indicated that SR scores for the *Imm-Ext* (*n* = 40) group were significantly larger (less phototactic suppression) than for the *Paired* group, for both start (Figure 2A; *F*_(1,58)_ = 10.90, *p* = 0.001) and finish (Figure 2B; *F*_(1,58)_ = 7.91, *p* = 0.004) latency behavior.

Pairwise comparisons between *Paired* and *Imm-Ext* groups at each RI revealed that the extinction-produced reduction of phototactic suppression was only partial at the 2-h RI, but clearly evident and near complete thereafter. SR scores of the *Imm-Ext* group were significantly larger than the Paired group in terms of start latency, but not finish latency, at the 2-h RI (*p*’s = 0.046, 0.072, respectively). Significant differences for both measurements were found at the remaining RIs. *Imm-Ext* animals had significantly larger SR scores than *Paired* animals for both start and finish latency behavior, respectively, at the 24- (*p*’s = 0.023, 0.027), 48- (*p*’s = 0.002, 0.003), and 72-h (*p*’s = 0.002, 0.023) RIs (Figures 2A,B).

Extinction training returned phototactic behavior back to *Random* control levels. This effect was partially complete at the 2-h RI, but complete by the 24-h RI. *Imm-Ext* animals displayed SR scores that were indistinguishable from *Random* control animals at the start (Figure 2A; *F*_(1,52)_ = 0.80, *p* = 0.189), but not finish latency behavior across all four RIs (Figure 2B; *F*_(1,52)_ = 3.10, *p* = 0.042). Analysis of SR scores at each RI revealed that the finish latency difference was attributable to group differences at only the 2-h RI. *post hoc* analysis at each RI failed to reveal significant differences between the groups for start, but not finish latency behavior, at the 2- (*p*’s = 0.152, 0.046, respectively). No significant differences for start and finish latency SR scores were detected for the 24- (*p*’s = 0.297, 0.205), 48- (*p*’s = 0.175, 0.083), and 72-h (*p*’s = 0.075, 0.061) RIs. This evidence indicates that extinction training given shortly after associative conditioning reversed the behavioral effects of paired conditioning by the 24-h RI and phototactic suppression did not spontaneously recover at any RI.

The above comparisons between *Paired* and *Imm-Ext* animals represent the pooled results of three separate and independent replications that were conducted over a six-month period from multiple batches of animals. Separate analyses indicated that the major conclusions drawn from the pooled results were confirmed: (1) at a qualitative level in all three replications; and (2) quantitatively (i.e., statistically significant differences) in two of three replications. For example, in Replication #2, SR scores of *Paired* (*n* = 15) animals were significantly smaller than *Imm-Ext* (*n* = 20) animals across all four RIs for both start (*F*_(1,33)_ = 4.17, *p* = 0.025) and finish (*F*_(1,33)_ = 6.96, *p* = 0.007) latency behavior. Similarly, in Replication #3, *Paired* (*n* = 12) animals showed greater phototactic suppression than *Imm-Ext* (*n* = 13) animals at all four RIs for both start (*F*_(1,23)_ = 11.26, *p* = 0.002) and finish (*F*_(1,23)_ = 10.04, *p* = 0.002) latency behavior.

### One day of paired conditioning produces phototactic suppression

The data above indicate that extinction training given shortly (15 min) after the end of paired conditioning attenuates pairing-produced phototactic suppression through a process that appears to reverse and/or antagonize the original associative memory. An alternative interpretation is that extinction training produces these effects on phototaxis by disrupting the consolidation of associative conditioning (Cain and Barad, 2003, but see Quirk, 2002). This explanation relies on the premise that associative conditioning has not been substantially consolidated by the end of the second day of paired conditioning when extinction training is given, and thus is susceptible to disruption.

Studies from our lab have previously shown that a single training session of 50 paired light-rotation presentations results in significant phototactic suppression measured 24 h post conditioning (Farley et al., 1997; Figures 1, 6). In these earlier studies, we also compared the extent of phototactic suppression (measured at the 24-h RI) produced by 50, 100, or 200 paired conditioning trials (i.e., 1, 2, or 4 consecutive daily training sessions). Not surprisingly, there was a statistically significant tendency for suppression to increase with the number of training trials/sessions (Farley et al., 1997; Figure 6). However, the suppression score after just 50 paired trials (0.33) was 97% of that exhibited after 200 trials (0.31), indicating that a single conditioning session of 50 pairings is more than sufficient to produce robust, consolidated memory. Other groups working with *H.c.* have also reported that a single session of 50 pairings of light and high-speed rotation produced significant phototactic suppression at a 24-h RI, although the training procedures were slightly different than ours (relatively brief (2–3 s), rather than prolonged duration (30 s), light and rotational stimuli) (Matzel et al., 1990).

**Figure 3:**
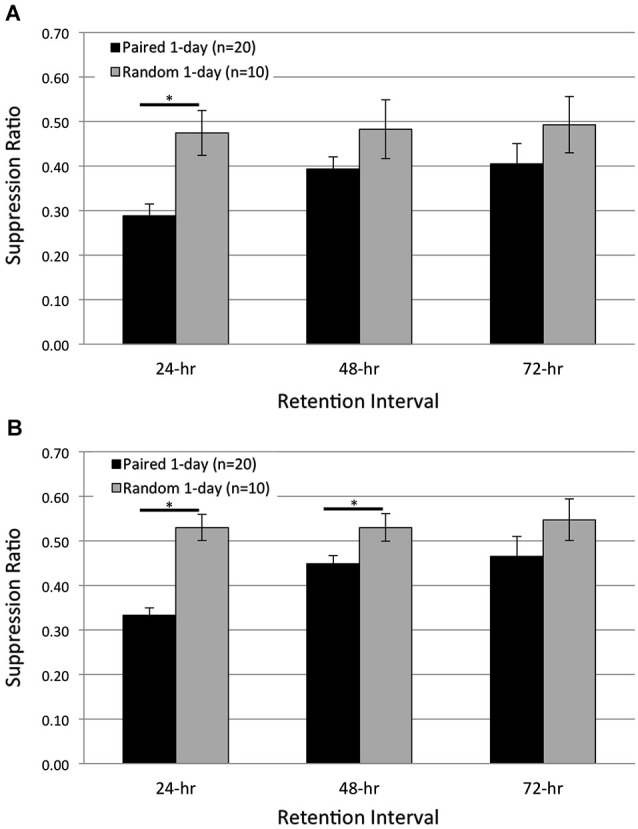
**One day of paired conditioning produces significant phototactic suppression compared to random control conditions.** Summary data for start **(A)** and finish **(B)** latency suppression ratio (SR) scores measured at three different RIs post-acquisition. Animals given one session of 50 paired conditioning trials (*Paired 1-day* group) showed significant phototactic suppression (i.e., smaller SR scores) compared to control animals given one session of 50 random presentations of light and rotation (*Random 1-day* group), for both the start and finish latency measure. *Paired 1-day* animals displayed significantly greater phototactic suppression than *Random 1-day* animals for start latency behavior at the 24-h RI, and significantly more suppression for finish latency behavior at the 24- and 48-h RIs. These data indicate that the memory for associative conditioning has been consolidated 24 h following one session of paired conditioning. Error bars are ± S.E.M and significant *p* values (*p*’s < 0.05) are denoted by an asterisk.

**Figure 4:**
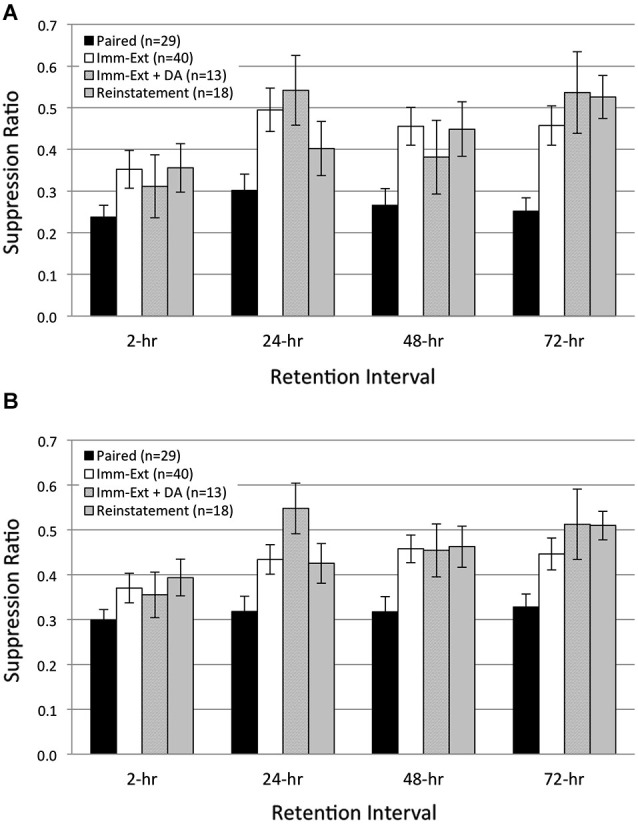
**Additional rotation stimuli failed to reinstate extinguished phototactic suppression.** Average start **(A)** and finish **(B)** latency suppression ratio (SR) scores measured at four different RIs post-acquisition. Animals (*Reinstatement* group) given extinction training followed by five additional rotation stimuli prior to the 2-h RI, and another five rotation stimuli prior to the 24-h RI, did not show reinstatement of extinguished phototactic suppression produced by paired conditioning. *Reinstatement* animals exhibited phototactic behavior that was indistinguishable from animals given immediate extinction training (*Imm-Ext* group) at all four RIs for both start **(A)** and finish **(B)** latency behavior. This result was also found when controlling for the additional 15 min period of dark adaptation given to *Reinstatement* animals; SR scores for *Immediate Extinction* animals given extra dark adaptation (*Imm-Ext + DA* group) were no different from *Reinstatement* group SR scores for both start **(A)** and finish **(B)** latency behavior. Error bars are ± S.E.M.

**Figure 5:**
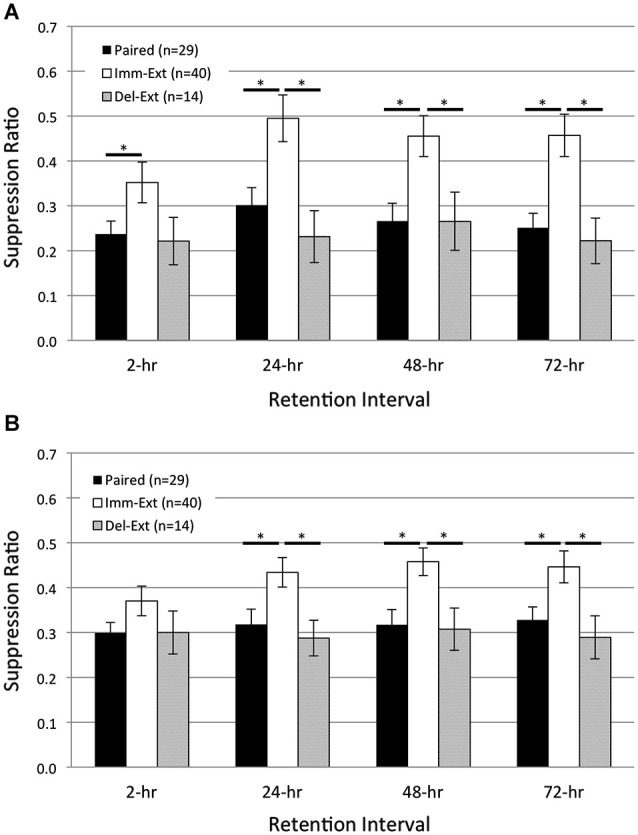
**Delayed behavioral extinction did not attenuate pairing-produced phototactic suppression.** Summary data for start **(A)** and finish **(B)** latency suppression ratio (SR) scores measured at four different RIs post-acquisition. Animals given delayed extinction training 23 h after the conclusion of paired conditioning (*Del-Ext* group) showed significant phototactic suppression (i.e., smaller SR scores) compared to animals given immediate extinction training (*Imm-Ext* group) 15 min after paired conditioning, for both the start and finish latency measure at the 24-, 48-, and 72-h RIs. *Del-Ext* animals were no different than *Paired* animals (not exposed to extinction) at any RI, for either the start or finish latency measure. *Imm-Ext* animals displayed significantly less phototactic suppression than *Paired* animals for start latency behavior at the 2-h RI, and less suppression for both start and finish latency behavior at the 24-, 48-, and 72-h RIs. Error bars are ± S.E.M and significant *p* values (*p*’s < 0.05) are denoted by an asterisk.

**Figure 6:**
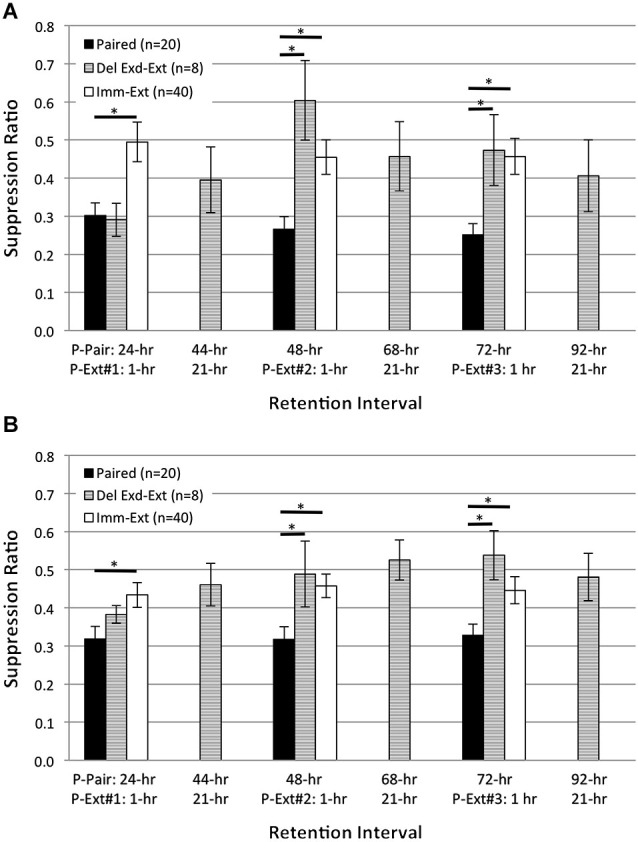
**Extended delayed extinction training attenuates pairing-produced phototactic suppression**. Summary data for start **(A)** and finish **(B)** latency suppression ratio (SR) scores measured at six different RIs following the end of acquisition training. X-axis labels show the RI time post paired training (P-Pair) or post extinction training (P-Ext) for three different extended extinction sessions (50 light-alone presentations; P-Ext#1-3). Animals given delayed extended extinction training (*Del Exd-Ext* group) 23 h after the conclusion of paired conditioning showed significant phototactic suppression (i.e., smaller SR scores) at the 24-h RI and were no more suppressed than animals given paired conditioning without extinction (*Paired* group), for both start and finish latency. On the next day, *Del Exd-Ext* animals were given a behavior test to assess the persistent effects of the previous day of extended extinction training (44-h RI). At this time, phototactic start and finish latencies were very similar to baseline, pre-training values (SR scores of ~0.5). Animals then received a second session of extended extinction training followed by a behavior test (48-h RI). By the 48-h RI, *Del Exd-Ext* animals showed SR scores that were significantly larger (i.e., less suppression) than *Paired* animals, for both start and finish latency measures. The following day, animals were given another behavior test (68-h RI), and no significant suppression of phototaxis was observed. Animals were then given a third session of extended extinction training followed by another behavior test (72-h RI). At the 72-h RI, *Del Exd-Ext* animals still displayed significantly less suppression than *Paired* animals. The following day, *Del Exd-Ext* animals were given a final behavior test (92-h RI), and continued to show no significant phototactic suppression, i.e., no evidence for spontaneous recovery of phototactic suppression was observed. SR scores of *Del Exd-Ext* animals were not significantly different from animals given immediate extinction training (*Imm-Ext* group) for both the start and finish latency measure at 24-, 48-, and 72-h RIs. Error bars are ± S.E.M and significant *p* values (*p*’s ≤ 0.05) are denoted by an asterisk.

Despite this evidence, it is possible that the conditions used in the present extinction experiments (reduced intensities of housing, training, and testing lights) might be different enough from previous studies that the memory produced by a single session of 50 pairings was weaker and/or more labile than observed previously. So, to directly address this concern, we compared phototactic suppression for Paired (*n* = 20) and Random control (*n* = 10) animals, 24, 48, and 72 h following a single training session of 50 of the appropriate trials (*Paired 1-day* and *Random 1-day* groups; Figure 3). If *Paired 1-day* animals exhibit significant phototactic suppression compared to *Random 1-day* animals, this would indicate that 1 day of paired conditioning produced sufficient consolidation of the associative memory to demonstrably impact behavior.

Consistent with this prediction, *Paired 1-day* animals showed substantial suppression of both start- and finish-latencies at the 24-h RI, which weakened somewhat by the 48-, and 72-h test (Figure 3). In contrast, *Random 1-day* animals showed no suppression at any time. When averaged across all three RIs, the suppression of start latency behavior exhibited by *Paired 1-day* animals was significantly greater than that of *Random 1-day* controls (*F*_(1,28)_ = 6.35, *p* = 0.009; Figure 3A). Planned pairwise comparisons at each RI revealed that *Paired 1-day* animals were significantly more suppressed than controls at the 24-h RI (*p* = 0.001), but no differences were detected at the 48- and 72-h RIs (*p*’s = 0.077, 0.134, respectively). Similar results were found for finish latency data (Figure 3B). On average, *Paired* *1-day* animals were significantly more suppressed than *Random* *1-day* controls (*F*_(1,28)_ = 17.09, *p* = 0.000). Pairwise analysis at each RI indicated significant differences between the groups at the 24- (*p* = 0.000) and 48-h (*p* = 0.013) RIs, but not at the 72-h (*p* = 0.133) RI (Figure 3B).

Thus, these data indicate that with present training and testing conditions, memory for the effects of a single session of 50 paired training trials was strong and stable enough to suppress phototaxis 24 h later, and was moreover indistinguishable at that time from that of animals receiving two full training days. However, it seems very likely that the second training day further strengthened this memory, since animals that received 2 days of training showed more prolonged significant suppression of phototaxis (Figure 2). These two-day (100 trial) animals showed significant suppression, for both start and finish latency results, at the 48- and 72-h RIs, in addition to the 24-h RI, in contrast to the results of the present (single session) 50 trial animals.

Therefore, we think it highly likely that a single session of 50 pairings produced sub-asymptotic learning and memory, and that the second acquisition day of 50 pairings produced some additional strengthening of phototactic suppression. Further, it is possible that extinction training administered ~15 min after the end of the second acquisition day might have disrupted consolidation of the second day’s training effects. However, there’s no good reason to believe that the effects of the 1st day of acquisition training were still undergoing consolidation at this time. Hence, it is difficult to interpret the success of the relatively immediate extinction training in reversing phototactic suppression (when given shortly after the second acquisition day) as having resulted from its interference with consolidation of memory from acquisition training on *both* the 1st and 2nd day, especially since delaying the delivery of extinction (25 LSs) until 23 h after the second acquisition session (analogous to delayed presentation of extinction after the first session) failed to reverse/weaken phototactic suppression.

### Additional US presentations do not reinstate extinguished learning

In both vertebrate (Rescorla and Heth, 1975) and invertebrate (Sangha et al., 2003; Plath et al., 2012) preparations, additional US presentations following extinction training can reinstate an extinguished CR. Because reinstatement is commonly used as evidence for the persistence of associative memories after extinction, we used a reinstatement procedure to further probe the presence of associative learning in *H.c.* after extinction training. We predicted that reinstatement training would be ineffective in restoring the extinguished CR of phototactic suppression in *H.c.*. As in the first experiment, animals were given immediate extinction training (15 min acquisition-extinction interval) followed by five unsignaled US (rotation) presentations ~30 min prior to the 2-, and 24-h RIs (*Reinstatement* group). We chose five rotation stimuli as the reinstatement protocol because this number of USs is sufficient to produce detectable changes in phototaxis when paired with light (Farley, 1987b; Grover et al., 1987). Additionally, the small number of stimuli limited exposure to prolonged periods of darkness and precluded any non-specific reductions in phototaxis that can occur due to multiple, consecutive dark adaptation periods (Grover et al., 1987). In line with our expectations, five additional rotation stimuli failed to reinstate extinguished phototactic suppression at any RI (Figure 4) and no differences in phototactic behavior were evident between *Reinstatement* (*n* = 18) animals and animals only given extinction training (*Imm-Ext* group). Across all RIs, SR values of the *Reinstatement* group were not statistically different from the *Imm-Ext* group, both in terms of start (Figure 4A; *F*_(1,61)_ = 0.17, *p* = 0.339) and finish (Figure 4B; *F*_(1,61)_ = 0.91, *p* = 0.172) latency behavior.

Because *Reinstatement* animals received an additional 15 min period of dark adaptation prior to each of the two sets of five rotation stimuli, we next assessed the possibility that additional dark adaptation periods might have affected phototactic behavior and obscured any reinstatement effects produced by the additional rotation stimuli. A comparison of SR values between the *Reinstatement* group and a separate group of *Imm-Ext* animals that received an additional dark adaptation period (*Imm-Ext + DA* animals, *n* = 13) without additional rotation stimuli found no significant differences for both start (Figure 4A; *F*_(1,29)_ = 0.01, *p* = 0.453) and finish (Figure 4B; *F*_(1,29)_ = 0.14, *p* = 0.357) latency behavior. This demonstrated that the additional dark adaptation period did not produce an independent facilitative effect on the phototactic behavior of *Reinstatement* animals that might have counteracted any suppressive effects of the additional rotation stimuli. Overall, these results indicated that the administration of five additional rotational stimuli prior to the 2-, and 24-h RIs was not sufficient to reinstate extinguished phototactic suppression.

### Delayed behavioral extinction fails to reverse paired conditioning

Our behavioral experiments reported above (Figures 2, 4) showed that extinction training given relatively immediately (15 min) after paired conditioning effectively reversed the pairing-produced suppression of phototaxis by the 24-h RI. Extinction-produced reversal of conditioned behavior and the corresponding reversal of correlated cellular changes have been reported to occur using relatively short (1 h) acquisition-extinction intervals (Mao et al., 2006; Myers et al., 2006). However, because this interval does not always produce reversal-like effects (i.e., temporary reductions of the CR, Maren and Chang, 2006; Woods and Bouton, 2008), we next investigated whether extinction-produced reversal of behavioral CRs in *H.c.* required short extinction-acquisition intervals. Therefore, we repeated the previous behavioral conditioning experiments using 2 days of paired conditioning as before, but delayed the administration of extinction training until 23 h after the end of paired training (23 h was chosen to ensure the 24-h RI test was held constant).

The phototactic behavior of *Delayed Extinction* *(Del-Ext)* animals was then compared to the behavior of *Paired* (no extinction) and *Immediate Extinction* *(Imm-Ext)* animals reported above for the 2-, 24-, 48-, and 72-h RIs (note that at the 2-h RI, *Del-Ext* animals had not received any extinction training). If delaying extinction training by 23 h provides sufficient time for consolidation of paired conditioning, and thus makes the memory more resistant to extinction, we would expect *Del-Ext* animals to display suppressed phototactic behavior that is similar to *Paired animals*, and more suppressed than *Imm-Ext* animals. As expected, delayed extinction failed to reverse pairing-produced phototactic suppression. *Del-Ext* animals were generally slower, for both start and finish latencies, than *Imm-Ext* animals, and this phototactic suppression was indistinguishable from *Paired* animal behavior.

A one-way ANOVA of start latency SRs across all four RIs revealed that SRs of *Del-Ext* (*n* = 14) animals were significantly smaller (i.e., greater suppression) than for *Imm-Ext* (*n* = 40) animals (*F*_(1,52)_ = 9.42, *p* = 0.002) and no different from *Paired* (*n* = 29) animals (*F*_(1,41)_ = 0.43, *p* = 0.258; Figure 5A). Planned pairwise comparisons between the two extinction conditions at each RI revealed that *Del-Ext* animals were generally slower than *Imm-Ext* animals, but this effect was more apparent after the 2-h RI; no significant differences were found for start latency SRs at the 2-h RI (*p* = 0.061), but significant differences were evident at the 24- (*p* = 0.004), 48- (*p* = 0.016), and 72-h (*p* = 0.005) RIs. Additional comparisons between *Del-Ext* and *Paired* animals indicated that delayed extinction (unlike immediate extinction) did not reverse pairing-produced phototactic suppression. The suppressed phototaxis displayed by *Del-Ext* animals was not significantly different from *Paired* animals at any of the four RIs (*p*’s = 0.393, 0.155, 0.495, 0.314; Figure 5A).

Similar results were found for finish latency data (Figure 5B). A one-way ANOVA of finish latency SRs across all four RIs indicated that SRs of *Del-Ext* animals were smaller than *Imm-Ext* animals (*F*_(1,52)_ = 7.63, *p* = 0.004) and no different from *Paired* animals (*F*_(1,41)_ = 0.28, *p* = 0.301; Figure 5B). Planned pairwise comparisons between the two extinction groups at each RI found no significant differences at the 2-h RI (*p* = 0.132), but did find differences at the 24- (*p* = 0.009), 48- (*p* = 0.007), and 72-h (*p* = 0.011) RIs, indicating that the *Del-Ext* group displayed slower phototaxis than the *Imm-Ext* group. No significant differences in SRs were found between *Paired* and *Del-Ext* groups at any of the four RIs (*p*’s = 0.500, 0.286, 0.436, 0.235; Figure 5B). Thus, extinction training delayed by 23 h was ineffective at attenuating pairing-produced phototactic suppression. These data support the conclusion that extinction-produced reversal of associative memories occurs within a limited time window.

### Extended delayed extinction training abolishes phototactic suppression

The failure of 25 delayed extinction trials to diminish phototactic suppression whatsoever was striking, especially since the same trials administered 15 min following the end of acquisition training completely abolished phototactic suppression. We therefore wondered if the robust phototactic suppression observed 24 h after 2 days of paired training was completely impervious to extinction training, or whether a greater number of trials and/or learning sessions (two variables which generally increase the strength of most forms of learning) might reveal some effects of additional extinction training.

We therefore conducted extended extinction training in which we repeated the initiation of extinction 23 h after the second day of 50 light-rotation pairings as before, but gave 50 rather than 25 non-reinforced LSs. This 50 trial extinction session was then repeated on two subsequent days, for a total of 150 extinction trials administered across three successive training days/sessions (see Table 1 for training and behavioral testing schedule). Tests of phototactic behavior were given ~1 h following the first 50 extinction trials (24 h following the end of the previous day’s 50 light-rotation pairings), both 1 h before and after the second and 3rd block of 50 extinction trials (at RIs 44-, 48-, 68-, and 72-h, measured relative to the end of acquisition training), and for a final time 24 h after the third block of extinction trials (i.e., 92-h following the end of acquisition training). The tests at the 44-, 68-, and 92-h RIs allowed an assessment of cumulative memory for the effects of the previous block(s) of 50 extinction trials, measured 24 h following the end of the previous block, and also constituted tests for “spontaneous recovery” from any extinction (weakened phototactic suppression) observed following the conclusion of extinction training on the preceding day. The tests at the 24-, 48- and 72-h RIs allowed an assessment of any decrements in response to suppression that were evident 1 h following the preceding block of 50 extinction trials.

**Table 1:** **Training and behavioral testing (Test) schedule for animals given delayed (24 h) extended extinction training (Extended Ext.) that comprised three sessions of 50 light-alone presentations**.

Training and Behavioral Testing Schedule								
Day:	Time:	9 am	10 am	11 am	12 pm	1 pm	2 pm	3 pm
1					Paired Train			
2					Paired Train			
3				Extended Ext.			Test (24-h RI)	
4			Test (44-h RI)	Extended Ext.			Test (48-h RI)	
5			Test (68-h RI)	Extended Ext.			Test (72-h RI)	
6			Test (92-h RI)					

*The behavioral test retention interval (RI) times indicate the number of hours post acquisition training. The 6.5 h light portion of the 6.5/17.5 light/dark cycle lasted from 9:00 am to 3:30 pm*.

As depicted in Figure 6, animals tested 1 h after the first block of 50 extinction trials (24-h RI, measured relative to the end of paired acquisition) showed start and finish latency suppression scores that were not significantly different from *Paired* animals tested at the same time who had not experienced extinction. This replicates the previous results (Figure 5) on the ineffectiveness of 25 delayed extinction trials in weakening suppressed phototaxis when extinction was assessed shortly following extinction, and also indicates that 50 extinction trials are no better than 25 at this time. However, on the next day (44-h RI; 21 h after the end of the first block of 50 extinction trials), animals that had received extended extinction showed weakened suppression scores for both start and finish latency. Four h later (48-h RI; 1 h after the end of the second block of 50 extinction trials), these same extinction animals now showed further weakened phototactic suppression scores for both start and finish latency, and in fact were not at all suppressed at this time. An isochronal comparison with the results from the *Imm-Ext* animals (results from Figure 5 have been replotted here in Figure 6) indicates that the two extinction treatments had produced comparable abolition of phototactic suppression at this time. The same pattern of results was repeated again on the next day. Animals that had received a total of 100 extinction trials, distributed across two extinction sessions, showed no evidence of phototactic suppression for either start or finish latencies (68-h RI). An additional 50 extinction trials (for a total of 150), failed to change the pattern when animals were tested ~1 h after extinction (72-h RI). The final test of phototactic behavior, at the 92-h RI (21 h following the third block of 50 extinction trials), continued to show no evidence of phototactic suppression for start and finish latency, indicative of no spontaneous recovery at this time.

Statistical analyses supporting these conclusions were of two types: (1) within-group, matched-sample comparisons of suppression ratios for the *Del Exd-Ext* animals at 24-h vs. 48-h RIs, and 24-h vs. 72-h RIs; (2) mixed-design repeated measures ANOVA for the results of *Paired vs. Imm-Ext vs. Del Exd-Ext*. For the *Del Exd-Ext* animals, start latency SR scores (Figure 6A) were significantly different for 24-h vs. 48-h RIs and for 24-h vs. 72-h RIs (*t*_(7)_’s = 4.27, 2.37, *p*’s = 0.002, 0.025, respectively), but not for 24-h vs. 92-h RIs (*t*_(7)_ = 1.79, *p* = 0.058). Finish latency SR scores (Figure 6B) were significantly different for 24-h vs. 72-h RIs and for 24-h vs. 92-h RIs (*t*_(7)_’s = 2.46, 1.99, *p*’s = 0.022, 0.043, respectively), but not for 24-h vs. 48-h RIs (*t*_(7)_ = 1.22, *p* = 0.132). Mixed-design ANOVA for the results of *Paired vs. Imm-Ext vs. Del Exd-Ext* at 24-, 48-, and 72-h RIs revealed a main effect of conditioning treatment for start (*F*_(2,74)_ = 9.29, *p* = 0.000; Figure 6A) and finish (*F*_(2,74)_ = 6.69, *p* = 0.001; Figure 6B) latency, with *Paired* animals showing significantly greater suppression than the two extinction treatments, when averaging across the different RIs. Planned comparisons revealed that *Paired* animals were more suppressed than *Imm-Ext* at 24-, 48-, and 72-h RIs, respectively, for start (*p*’s = 0.001, 0.007, 0.002) (Figure 6A) and finish (*p*’s = 0.021, 0.006, 0.024; Figure 6B) latency, and were more suppressed than *Del Exd-Ext* animals at the 48- and 72-h RIs, but not at the 24-h RI, respectively, for start (*p*’s = 0.003, 0.050, 0.500) and finish (*p*’s = 0.044, 0.015, 0.500) latency. Comparisons between both extinction groups failed to find any significant differences at any RIs for both start (*p*’s = 0.065, 0.227, 0.500) and finish (*p*’s = 0.500, 0.500, 0.366) latency.

In summary, we found that delayed extinction training was capable of reversing phototatic suppression if a large number of extinction trials (150 total) were administered, across multiple successive extinction sessions (*Del-Exd-Ext* results in Figure 6). Most (but not all) of the reversal appeared to have occurred after the first 50 LSs, with the subsequent 100 LSs contributing less. The ability of 50, but not 25, delayed LSs to attenuate phototactic suppression further indicates that extinction-produced reversal of the memory for pairings of light and rotation in *H.c.* is not limited to a brief post-acquisition window of time, but instead extends for at least 24 h following original acquisition.

Some ambiguity surrounds the correct interpretation of our finding that the first session of delayed extended extinction training (50 LSs) failed to reverse phototactic suppression when animals were tested relatively soon (~1 h) after the conclusion of the first extinction session, whereas some reversal was observed 21 h later and complete reversal was apparent following the subsequent (second and 3rd) extinction sessions. The simplest and most straightforward explanation of this pattern is that consolidation and expression of extinction memory was time dependent, requiring more than an hour for its effects to be reflected in tests of phototaxis. Alternatively, weakening of phototactic suppression by the 1st session of extinction training may have been masked/opposed at the 1-h (post-extinction) RI by non-associative locomotor suppressive effects of extinction training (e.g., extended confinement in training tubes). Any such effects would be expected to have dissipated 21 h later (44 h following conclusion of second day of paired acquisition training), with the result that weakened phototactic suppression was now evident. This latter interpretation seems unlikely, given that no phototactic suppression was ever observed at the subsequent 1-h tests following the second and 3rd extinction sessions (i.e., P-Ext #2-1 h and P-Ext#3-1 h results in Figure 6).

### Extinction training reduces the enhanced excitability of Type B photoreceptors due to pairings of light and rotation

**Figure 7:**
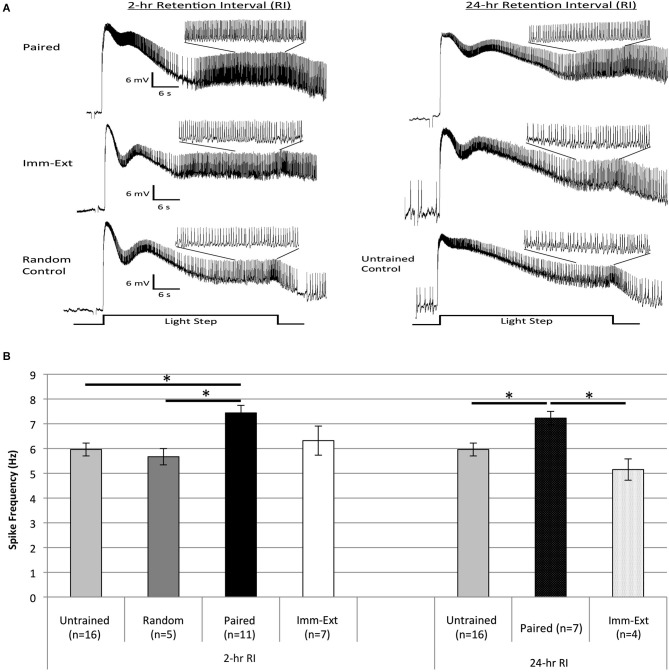
**Extinction training reduced the enhanced B cell spike frequency produced by paired conditioning.** **(A)** Representative light responses recorded from either paired-trained (top trace), immediate extinction-trained (middle trace), or control (bottom trace) Type B photoreceptors from isolated *H.c.* nervous systems. Light responses were obtained either 2 h (left) or 24 h (right) after the end of paired (or random) conditioning [2-, and 24-h RI]. Light-evoked generator potentials and spike frequencies are shown for the first 30 s light step (LS). Inset traces show expanded time scale for the last 10 s of the 30 s LS. Note the greater spike frequency of the *Paired* cell compared to control cells, indicative of associative learning. Extinction training given shortly after paired conditioning (*Imm-Ext* group) slightly reduced the pairing-produced increases in spike frequency at the 2-h RI. By the 24-h RI, the extinction-produced spike frequency reduction was greater and statistically significant. **(B)** Summary spike frequency data for trained cells and control cells during LSs 1 and 2. Spike frequencies were recorded over the last 10 s of each 30 s LS. Paired conditioning increased Type B cell spike frequency during LSs 1 and 2 above *Untrained* and *Random* (non-associative) control cells, which failed to differ. Immediate extinction *(Imm-Ext)* significantly reduced this increased spike frequency at the 24-h RI. No significant differences were found between *Imm-Ext* cells and control cells at either the 2-, or 24-h RIs. Error bars are ± S.E.M and significant *p* values (*p*’s < 0.05) are denoted by an asterisk.

Previous research in *H.c.* identified the Type B photoreceptors as a principal site of memory storage for associative conditioning (Crow and Alkon, 1980; Farley and Alkon, 1980, 1982; Farley et al., 1983), and as an important cellular correlate of extinction learning (Richards et al., 1984). The initial analysis of extinction effects on Type B cells (Richards et al., 1984) focused on alterations of the light-induced generator potentials of synaptically-isolated cells. These cells do not spike because of axotomy and are critical for establishing whether extinction-produced changes in B cell excitability reflect changes intrinsic to B cells. Here, we extended our approach to also include an analysis of the light-evoked spike frequencies of synaptically-intact B cells from *Paired*, *Imm-Ext*, and non-associative control (*Random* and *Untrained*) conditions, at two RIs (2-, and 24-h) for which behavioral tests of phototaxis were conducted (Figure 2). Animals were randomly selected from each of these conditions and prepared for recording. Spike frequencies were then measured during the final 10 s of two consecutive LSs. As with many previous reports (Farley and Alkon, 1982; Farley, 1987a), paired training increased spike frequencies (i.e., enhanced excitability) of Type B cells when recorded from 24-h post-conditioning. Extinction training administered immediately after the end of paired training (*Imm-Ext* animals) returned the increases in B cell excitability back to control levels, findings consistent with an extinction-produced reversal hypothesis (see Figure 7A for representative traces).

A one-way ANOVA of spike frequencies during LSs 1–2 for cells from *Paired* (*n* = 7), *Imm-Ext* (*n* = 4), and *Untrained* (*n* = 16) groups 24-h post conditioning found a main effect of training condition (*F*_(2,24)_ = 7.17, *p* = 0.002; Figure 7B, right). *Post hoc* tests revealed that Type B cells from *Paired* animals showed greater light-evoked spike frequencies (7.23 ± 0.27 Hz) than cells from *Untrained* control preparations (5.96 ± 0.26 Hz; *p* = 0.020). Further *post hoc* tests indicated that B cells from *Imm-Ext* animals spiked less frequently (5.15 ± 0.43 Hz) than those from *Paired* animals (*p* = 0.003), and were indistinguishable from *Untrained* cells (*p* = 0.203; Figure 7B, right).

When recorded 2 h post-conditioning, significant differences in spike frequencies were found between the *Paired* and control training conditions, but not between *Paired* and *Imm-Ext* conditions, although the same qualitative trend apparent at 24 h was evident at 2 h as well (Figure 7). A one-way ANOVA conducted for spike frequencies during LSs 1–2 for cells from *Paired* (*n* = 11), *Imm-Ext* (*n* = 7), *Untrained* (*n* = 16), and *Random* (*n* = 5) groups revealed a main effect of training condition (*F*_(3,35)_ = 4.70, *p* = 0.004; Figure 7B, left). *Post hoc* tests indicated that spike frequencies of *Paired* cells (7.44 ± 0.31 Hz) were significantly greater than *Untrained* (5.96 ± 0.26 Hz; *p* = 0.006) and *Random* (5.67 ± 0.33 Hz; *p* = 0.018) control cells. The two control conditions did not differ significantly (*p* = 0.500). Spike frequencies of *Imm-Ext* (6.32 ± 0.59 Hz) cells were slightly, though not significantly, lower than *Paired* cells (*p* = 0.139), and no different from *Untrained* (*p* = 0.500) and *Random* (*p* = 0.500) control cells (Figure 7B, left).

These results generally paralleled the behavioral changes observed for intact animals from the same treatment conditions, at both RIs. The pairing-produced increases in Type B cell spike frequency were not fully reversed by extinction training when measured 2 h after conditioning. However, the increases in excitability were abolished by extinction at the 24-h RI, indicating that the impact of extinction on cellular excitability takes time to develop (or to be expressed). These results further support the view that relatively immediate extinction training reverses not only the behavioral changes due to original acquisition (with no additional evidence of spontaneous recovery), but also opposes one of the fundamental processes of enhanced neuronal excitability (viz., enhanced spike frequency in B cells) that underlies the pairing-produced suppression of phototaxis (Farley et al., 1983).

## Discussion

The present results are consistent with the hypothesis that appropriately timed extinction training (non-reinforced presentations of light) reverses the behavioral and cellular effects of associative conditioning in *H.c.* We found that these effects occurred when a single session of extinction training (25 LSs) was administered 15 min, but not 23 h, after the conclusion of pairings of light and rotation. However, a much greater number of extinction trials (total of 150), distributed over three successive daily sessions, was able to abolish phototactic suppression, even if initiation of the first extinction session was delayed by 23 h post-acquisition training. Extinction training reduced conditioned behavior (phototactic suppression, the CR) back to baseline pre-conditioning levels. This was observed for two measures of phototaxis, start and finish latency. Extinguished behavior did not return up to 72 h after conditioning (failure to observe spontaneous recovery), nor after extinction-trained animals received additional unsignaled rotation (US) presentations (failure to observe reinstatement). These two results suggest that the memory for associative conditioning is absent following extinction (Richards et al., 1984). Our electrophysiological data further reinforced this conclusion and indicated that extinction training reversed enhanced Type B photoreceptor excitability, one of the critical cellular correlates of associative conditioning, when measured 24 h after acquisition training. Together, the behavioral and neurophysiological findings indicate that for *H.c.*, extinction training eliminates, or enduringly prevents the detection of, associative memories on the cellular and behavioral levels.

### Extinction reverses conditioned phototactic suppression, no evidence of spontaneous recovery or reinstatement

Our finding that extinction reversed conditioned phototactic suppression without spontaneous recovery replicates earlier *H.c.* extinction research (Richards et al., 1984), and extends that analysis to two additional retention intervals (RIs; 48- and 72-h) post learning acquisition. The present study used the same behavioral dependent measure of finish latency reported by Richards et al. (1984), along with the additional measure of start latency, indicating that extinction-produced reversal of conditioned behavior is not limited to one behavioral measure. The absence of phototactic suppression after extinction, combined with the lack of spontaneous recovery and the failure to observe reinstatement, suggest that the associative memory is not present, at least in its original form, after extinction.

Additional tests for “renewal” of conditioning (i.e., the failure of extinction to generalize from one context to another, observed as the return of the CR when the CS is tested in a context different from that used during extinction training) were not attempted here because of uncertainty surrounding the possibility of context-specific phototactic conditioning with *H.c.* There is little precedent from previous research with *H.c.* for thinking that the standard light-rotation pairing-produced suppression of phototaxis varies appreciably with the presence or absence of additional, static, exteroceptive cues (e.g., chemosensory cues, substrate texture) that might be used to experimentally define a conditioning “context.” Further, attempts to demonstrate contextual-conditioning to chemosensory CSs with *H.c.* (using rotation as the US) have been notoriously difficult and, on the whole, unsuccessful (Jin et al., 2004). Although it is possible that the associative memory was present, but not detected, following extinction, this possibility seems unlikely. Our behavioral measures are able to detect learning-produced changes in phototaxis that arise from as few as 2–5 pairings of light and rotation (Farley, 1987b; Farley and Alkon, 1987; Grover et al., 1987).

The absence of both spontaneous recovery and reinstatement of phototactic suppression after extinction implies that the original associative memory has been erased, or at the very least substantially weakened. However, there are two alternative interpretations of our (behavioral) results that cannot be completely dismissed. First, as mentioned earlier in justifying the “1 day of paired conditioning experiment” (Section 1 day of paired conditioning produces phototactic suppression), the memory formed by just a single session of 50 paired trials (Figure 3) appeared weaker than that resulting from 2 days of training (i.e., 100 paired trials; Figure 2), in that it showed more rapid forgetting. Thus, it is possible to argue that the extinction trials administered relatively immediately (15 min) after the second day of training in some way re-activated memory from the previous day, allowing for modification of its content, while simultaneously disrupting consolidation of memory stemming from the second day of 50 paired trials. According to this scenario, the erasure/reversal of memory from 2 days of paired training, due to relatively immediate extinction trials administered after the second day, reflected a combination of “reconsolidation update” and “disruption of consolidation effects.” However, it is unclear why 25 LSs would be able to reactivate and modify the content of a relatively weak memory when given 24 h following the end of the first training day, while the same 25 LSs administered 24 h following the second training day were unable to reactivate and modify the content of the cumulative memory resulting from both days of paired training. A second alternative account of our results posits that successful extinction training produced its apparent reversal of initial acquisition memory through the occurrence of active inhibitory learning (Pavlov, 1927; Rescorla and Cunningham, 1978) that masked/opposed the expression of phototactic suppression.

*H.c.* readily exhibit CI learning (Britton and Farley, 1999; Farley et al., submitted), and because *H.c.* CI learning and extinction both share the involvement of protein phosphatase 1, PP1 (Cavallo et al., submitted; Farley et al., submitted) and arachidonic acid (AA)/12-lipoxygenase (12-LOX) metabolite (Walker et al., 2010) signaling pathways, this alternative warrants further serious study. However, if inhibitory learning was produced by extinction, this new learning might be expected to decay more quickly than the original associative memory (see Myers and Davis, 2002 for review), and therefore spontaneous recovery of phototactic suppression at later RIs might have been expected to occur, contrary to what we observed. Additional studies with *H.c.* will be necessary to determine if: (1) the extinguished CS (light) has acquired CI properties (i.e., appropriate summation test results with chemosensory CS+’s), or merely lacks conditioned excitatory value; and (2) whether the same CS can simultaneously be both a conditioned excitor and a conditioned inhibitor.

Finally, our observation that multi-trial, multi-session, extended extinction training was able to reverse the effects of 2 days of paired training (even when initiation of extinction training was delayed by 24 h following the end of acquisition) is noteworthy, especially since no evidence of spontaneous recovery was observed on the day following the end of extinction training (92-h RI in Figure 6). Like the results for extinction training involving just a single session of 25 LSs, administered 15 min after the end of acquisition training, the simplest and most parsimonious account of both patterns is that extinction reversed/altered the originally consolidated memory for pairings, irrespective of its strength. However, it is possible that distinct processes underlay the (apparent) reversal of memory by immediate- vs. delayed-, extended extinction training. Since we did not assess the possibility of spontaneous recovery following extended extinction at RIs past the 92-h RI, nor did we conduct reinstatement tests (or other recovery protocols) following the multi-session, extended extinction training, the possibility exists that two different memories competed for control of phototaxis (see Bouton, 2004) (original memory 1: “light is associated with rotation” vs. newer memory 2: “light is not associated with rotation” or, more generally, “light is presented alone, by itself”). In contrast, the reversal of what might have been a weaker form of memory, by a single session of 25 LSs given 15 min following original acquisition (Figure 2), that failed to show either spontaneous recovery at multiple RIs or reinstatement, may have predominantly reflected genuine modification (erasure) of original memory.

Assuming for the moment that the reversal of phototactic suppression by both extinction protocols reflected erasure-like modification of the original memory, it is interesting to consider possible reasons for the relative ease with which this result can be achieved in *H.c.* As noted earlier, erasure-like effects have also recently been reported for fear conditioning with (adult) rodents and humans, using reconsolidation paradigms. However, judging from the variability in outcome, the circumstances producing genuine “erasure” with rodents and humans appear to be considerably more circumscribed than is the case for *H.c.* Why might this be so? There are many possibilities, including the likelihood of obsessive ruminations and ongoing covert rehearsal processes of memories formed during aversive/fear conditioning paradigms with humans, and perhaps analogous (implicit) second-order conditioning processes in adult rodents that promote formation of S-R associations that are refractory to extinction, and US-devaluation/counterconditioning manipulations (see Rizley and Rescorla, 1972; Rescorla, 1980). Genuine and enduring erasure of fear memory in adult vertebrates may be particularly difficult to achieve precisely because of the multiple types of associative memories (S-S and S-R) that may participate in even simple CS-US fear conditioning paradigms. In contrast, erasure-like effects may be easier to obtain with *H.c.* because of the simpler associative structures (and correspondingly simpler neural circuits) that mediate learned suppression of phototaxis.

Higher-order conditioning and sensory preconditioning of phototactic suppression has been tested extensively in *H.c.*, and has not been observed (Farley et al., 1997). This might result in phototactic suppression being strongly dependent upon an S-S associative structure, and thus uncustomarily sensitive to erasure-like effects of extinction training. In a related vein, we (Richards et al., 1984) and many others (Groves and Thompson, 1970; Kamprath and Wotjak, 2004; Kamprath et al., 2006) have noted that aversive/fear conditioning paradigms with vertebrates often involve associative and non-associative learning processes that proceed in parallel, and interact to determine the strength of conditioned fear responses. For example, sensitization (defined as a generalized non-associative increase in responsiveness, following exposure to novel and/or noxious stimulation, e.g., the aversive stimuli typically used in fear conditioning) demonstrably occurs in many aversive conditioning situations and would be expected to contribute to the strength of the CR. Conversely, extinction training that involves repeated nonreinforced CS presentations may decrease the responsiveness to the CS in the relevant stimulus–response pathway(s) because of habituation-like processes (learning to ignore the CS), in addition to any modification(s) of associative linkages that result. Indeed, all three of the behavioral criteria (spontaneous recovery, reinstatement, context-dependency (renewal)) commonly used to argue for the general failure of erasure to occur during extinction are also cardinal features of habituation (Groves and Thompson, 1970).

Thus, if habituation is occurring within the neural circuit(s) that mediate acquisition/extinction of fear conditioning (in addition to the presumably activity-dependent forms of associative synaptic plasticity thought to be critical for CR-weakening, e.g., LTD at thalamic-lateral amygdala synapses), and further habituation is occurring at loci (e.g., thalamic nuclei, such as medial geniculate nuclei and posterior intralaminar nuclei in the case of auditory fear conditioning) that project to the more central site(s) of critical associative plasticity (e.g., lateral amygdala) (Gabriel et al., 1976; McEchron et al., 1995; Weinberger, 2011), then relatively short-term, labile habituation effects could be one important mechanism leading to diminished fear CRs during and shortly after extinction training. These changes might then preclude and “protect” the downstream sites of associative plasticity from undergoing more persistent “erasure” like changes. Hence, “spontaneous recovery” of the CR could then occur once a sufficient interval of no CS stimulation had elapsed. Similar arguments can in principle be advanced to explain reinstatement (arising from disruption of habituation, either dishabituation *per se* and/or independent sensitization due to US presentations) and renewal (habituation is also context dependent), as reflections of non-associative learning. If one provisionally grants the extensive commingling of non-associative and associative learning processes in vertebrate fear conditioning, then it is perhaps not so surprising that conventional extinction rarely results in genuine erasure. In contrast, persistent experience-induced modifications of phototaxis in *H.c.* (both suppression and enhancement) seem little influenced by either “habituation,” or persistent sensory adaptation, to light (see Richards et al., 1984; Farley, 1987a,b). This might lead to the expectation that manipulations that routinely disrupt habituation processes during extinction in vertebrate fear conditioning paradigms, and “unmask” original associative memories, would be without effect in *H.c.* That same absence of habituation of phototaxis might also be invoked to explain the relative ease with which reversal and erasure-like changes of original associative memory, due to pairings, can be produced in *H.c.*

### Extinction-produced reversal in other systems

Extinction-produced reversal of conditioned behavior is not unique to *H.c.* and has also been reported in a rat fear-potentiated startle paradigm using short acquisition-extinction intervals (Myers et al., 2006). Extinction training given 10 min, but not 72 h after learning acquisition produced robust reductions of conditioned fear without any evidence of spontaneous recovery or reinstatement (Myers et al., 2006). Similar results were reported with short (12 min) acquisition-extinction intervals using a rat conditioned freezing paradigm (Johnson et al., 2010, but see Schiller et al., 2008). Despite these examples, immediate extinction training does not always produce persistent reversal of the CR. Some research suggests that the ability of extinction to produce a lasting (i.e., reversal/erasure) or temporary reduction of the CR depends not on a single acquisition-extinction interval (e.g., short or long), but can include a critical “window” period. For example, in the honeybee (*A. mellifera*), spontaneous recovery of an extinguished olfactory memory was observed when the acquisition-extinction interval was either extremely short (1 min) (Sandoz and Pham-Delègue, 2004) or very long (24 h) (Stollhoff et al., 2005), but not when the interval was 10 min (Sandoz and Pham-Delègue, 2004). The complexity of these results suggests that the use of short acquisition-extinction intervals does not guarantee that extinction will alter the original memory and affect the CR.

Cellular and molecular evidence for a true extinction-produced “reversal/erasure” has generally been limited (Schwaerzel et al., 2002; Lin et al., 2003; Mao et al., 2006). However, recent research has uncovered evidence of an extinction-produced reversal/unlearning of fear-conditioning in juvenile (Kim and Richardson, 2007, 2008), as well as in adult rats (Quirk et al., 2010) and mice (Clem and Huganir, 2010). Thus, extinction training may recruit both “new” antagonistic learning, as well as “reversal” processes; under certain circumstances, a reversal of the original learned association may be the predominant mechanism.

In addition to the time at which extinction training is given, presumably other training variables are also important, such as the strength of the original memory (i.e., the number and temporal distribution of acquisition trials, the intensities of the CS and US, etc.), the developmental state of the organism (e.g., juvenile vs. adults, Kim and Richardson, 2008), and whether or not extinction training effectively activates “reconsolidation” processes (Monfils et al., 2009; Schiller et al., 2010). A unified account of the variables that favor an outcome of reversal/erasure following extinction training (or the inability to detect the original memory) remains an as yet unrealized goal.

### Extinction reverses cellular correlates of associative conditioning

Because associative conditioning increases Type B photoreceptor excitability, which is causally related to conditioned changes in behavior (Farley et al., 1983; Richards and Farley, 1987), we hypothesized that extinction training would not only prevent the detection of the associative behavioral changes, but also produce a corresponding reversal in B cell excitability. As predicted, extinction training given shortly (15 min) after paired conditioning reversed the pairing-produced increases in B cell spiking back to control levels. This reversal was partial at the 2-h RI, but apparently complete by the 24-h RI. The delayed expression of this spike frequency reversal is similar to the reduction in B cell spiking reported for CI learning, which was not present 2 h after conditioning, but was evident 24 h later (Walker et al., 2010). One factor that may account for the apparent delayed expression of both extinction- and CI-related changes in behavior and B cell excitability is the presence of competing, non-associative sources of phototactic suppression and enhanced B cell excitability owing to the preceding training period involving repeated US presentations. Previous research with *H.c.* has found that the pairing- and stimulus-specific reductions in phototaxis and enhancement of B cell excitability are often obscured by potent non-associative effects of training, principally the effects of repeated rotational/vestibular stimulation (Crow, 1983; Grover et al., 1987) and the sequelae of the activation of serotonergic and GABAergic systems. These neuromodulatory effects can persist for many minutes following the end of training, but have dissipated by 24 h post-acquisition.

The present results mirror our prior findings in *H.c.* (Richards et al., 1984), which showed that extinction training reversed the pairing-produced increases in light responses (both peak and steady-state generator potentials, SSPGs) and increases in resting input resistances, back to pre-training levels. The reversals of B cell excitability reported by Richards et al. (1984) occurred more quickly (2–4 h after training) than the present findings with spike frequency, which were only partially complete at the 2-h RI. The differences in time courses between our present findings and Richards et al. (1984) might reflect procedural differences between the two studies, differences in the persistence of the non-associative influences on B cell excitability between the two studies, and/or differences in the rates of signaling-cascades that underlie distinct aspects of B cell excitability. For example, learning-produced changes in light-evoked spiking and generator potentials appear to be differentially regulated by distinct classes of somatic K^+^ channels. The *I*_A_ current, by itself, is a critical regulator of spike frequency in other training conditions (e.g., CI learning; Farley et al., submitted; Walker et al., 2010) that leads to reductions in spike frequency. The expression of CI-produced decreases in SSGPs, however, depends upon increases in both *I*_A_ and *I*_delayed_/*I*_K-Ca_ (Farley et al., submitted), with the latter playing a larger role than the former. Thus, the putative molecular processes initiated by extinction training (e.g., phosphatase- and fatty acid-signaling) might increase the activity of calcium-activated K^+^ channels before that of the A-channels and thereby differentially affect the measurements of excitability at different rates. In addition, ongoing increases in reciprocal synaptic inhibition (and additional neuromodulatory influences) among Type B (and A) photoreceptors that are present in synaptically-intact spiking photoreceptors, may limit the expression of extinction-produced decreases in SSGPs. Removal of those influences, by elimination of the axodendritic process (as in Richards et al., 1984), may unmask the differences in SSGPs.

The reversal of learning-produced changes in cellular activity in response to extinction training is consistent with some findings in vertebrates. In rats, extinction of conditioned fear can be produced without spontaneous recovery and is associated with the reversal of the cellular changes elicited by fear acquisition, specifically the insertion of AMPA GluR1-containing receptors in the amygdala (Mao et al., 2006). Interestingly, the reversal of both cellular and behavioral changes occurred when extinction training was given 1 h after acquisition, but not when delayed by 24 h, which is similar to our behavioral data (*Imm-Ext vs. Del-Ext* groups) and *in vitro* extinction results (see accompanying paper, Cavallo et al., submitted). Mao et al. (2006) interpreted this reversal of learning-produced changes at both levels of analysis as evidence of true extinction-induced erasure. This conclusion is also consistent with extinction research using methods that alter or update a consolidated memory, which has correlated the permanent extinction of conditioned fear with the reversal of fear-induced synaptic strengthening and the subsequent removal of calcium-permeable AMPA receptors in mice brain slices that included the lateral amygdala (Clem and Huganir, 2010).

## Conclusion

Exposing *H.c.* to pairings of light and rotation (associative conditioning) produces characteristic changes in behavior (e.g., suppression of phototaxis) and neural excitability (e.g., increases in Type B cell light-evoked spike frequency). In this paper, we described: (1) an extinction paradigm that reversed the behavioral changes produced by paired training, without evidence of spontaneous recovery or reinstatement; (2) the reversal of pairing-produced increases in B cell excitability as a result of extinction; (3) the specific intervals between learning acquisition and extinction that allow extinction to alter the behavioral and cellular components of associative conditioning. Overall, these results are consistent with our early hypothesis (Richards et al., 1984) that extinction can reverse/erase both the behavioral and neural changes due to associative learning. However, our results obviously do not necessarily imply that *all* substrates of the original associative memory were erased or altered by extinction training. Multiple sites of enduring neuronal and synaptic plasticity due to pairings have been identified in *H.c.*, including Type A photoreceptors (Farley et al., 1990; Farley and Han, 1997) and Type I interneurons (Crow and Tian, 2002). Whether these substrates of memory are also affected by extinction training is an important issue for future studies. Whether extinction in *H.c.* produces an actual erasure of the original associative memory might depend on the level of analysis (i.e., behavior, circuit, or single neuron), as well as the sensitivity of the methods used to assess the issue.

## Conflict of interest statement

The authors declare that the research was conducted in the absence of any commercial or financial relationships that could be construed as a potential conflict of interest.
